# Stable stem enabled Shannon entropies distinguish non-coding RNAs from random backgrounds

**DOI:** 10.1186/1471-2105-13-S5-S1

**Published:** 2012-04-12

**Authors:** Yingfeng Wang, Amir Manzour, Pooya Shareghi, Timothy I Shaw, Ying-Wai Li, Russell L Malmberg, Liming Cai

**Affiliations:** 1 Department of Computer Science, University of Georgia, Athens, Georgia 30602, USA; 2 Department of Plant Biology, University of Georgia, Athens, Georgia 30602, USA; 3 Institute of Bioinformatics, University of Georgia, Athens, Georgia 30602, USA; 4 Center for Simulational Physics, University of Georgia, Athens, Georgia 30602, USA

## Abstract

**Background:**

The computational identification of RNAs in genomic sequences requires the identification of signals of RNA sequences. Shannon base pairing entropy is an indicator for RNA secondary structure fold certainty in detection of structural, non-coding RNAs (ncRNAs). Under the Boltzmann ensemble of secondary structures, the probability of a base pair is estimated from its frequency across all the alternative equilibrium structures. However, such an entropy has yet to deliver the desired performance for distinguishing ncRNAs from random sequences. Developing novel methods to improve the entropy measure performance may result in more effective ncRNA gene finding based on structure detection.

**Results:**

This paper shows that the measuring performance of base pairing entropy can be significantly improved with a constrained secondary structure ensemble in which only canonical base pairs are assumed to occur in energetically stable stems in a fold. This constraint actually reduces the space of the secondary structure and may lower the probabilities of base pairs unfavorable to the native fold. Indeed, base pairing entropies computed with this constrained model demonstrate substantially narrowed gaps of Z-scores between ncRNAs, as well as drastic increases in the Z-score for all 13 tested ncRNA sets, compared to shuffled sequences.

**Conclusions:**

These results suggest the viability of developing effective structure-based ncRNA gene finding methods by investigating secondary structure ensembles of ncRNAs.

## Background

Statistical signals in primary sequences for non-coding RNA (ncRNA) genes have been evasive [1-3]. Because single strand RNA folds into a structure, the most exploitable feature for structural ncRNA gene finding has been the secondary structure [4-6]. The possibility that folded secondary structure may lead to successful *ab initio *ncRNA gene prediction methods has energized leading groups to independently develop structure-based ncRNA gene finding methods [7,8]. The core of such a program is its secondary structure prediction mechanism, for example, based on computing the minimum free energy for the query sequence under some thermodynamic energy model [9-12]. The hypothesis is that the ncRNAs' secondary structure is thermodynamically stable. Nonetheless, stability measures have not performed as well as one might hope [13]; there is evidence that the measures may not be effective on all categories of ncRNAs [14].

A predicted secondary structure can be characterized for its fold certainty, using the Shannon base pairing entropy [15,16]. The entropy ∑*p_i,j _*log *p_i,j _*of base pairings between all bases *i *and *j *can be calculated based on the partition function for the Boltzmann secondary structure ensemble, which is the space of all alternative secondary structures of a given sequence; the probability *p_i,j _*is calculated as the total of Boltzmann factors over all equilibrium alternative structures that contain the base pair (*i*, *j*) [17]. As an uncertainty measure, the base pairing Shannon entropy is maximized when base pairing probabilities are uniformly distributed. A structural RNA sequence is assumed to have a low base pairing Shannon entropy, since the distribution of its base pairing probabilities is far from uniform. The entropy measure has been scrutinized with real ncRNA data revealing a strong correlation between entropy and free energy [18,19]. However, there has been mixed success in discerning structural ncRNAs from their randomly shuffled counterparts. Both measures perform impressively on precursor miRNAs but not as well on tRNAs and some rRNAs [14,18].

The diverse results of the entropy measuring on different ncRNAs suggest that the canonical RNA secondary structure ensemble has yet to capture all ncRNAs structural characteristics. For example, a Boltzmann ensemble enhanced with weighted equilibrium alternative structures has also resulted in higher accuracy in secondary structure prediction [19]. There is strong evidence that the thermodynamic energy model can improve its structure prediction accuracy by considering energy contributions in addition to those from the canonical free energy model [20,21]. Therefore, developing ncRNA structure models that can more effectively account for critical structural characteristics may become necessary for accurate measurement of RNA fold certainty.

In this paper, we present work that computes Shannon base pairing entropies based on a constrained secondary structure model. The results show substantial improvements in the Z-score of base pairing Shannon entropies on 13 ncRNA datasets [18] over the Z-score of entropies computed by existing software (e.g., NUPACK [23] and RNAfold [12,29]) with the canonical (Boltzmann) secondary structure ensemble and the associated partition function [22]. Our limited constraint to the secondary structure space is to require only canonical base pairs to occur in stable stems. The constrained secondary structure model is defined with a stochastic context-free grammar (SCFG) and entropies are computed with the Inside and Outside algorithms. Our results suggest that incorporating more constraints may further improve the effectiveness of the fold certainty measure, offering improved *ab initio *ncRNA gene finding.

## Results

We implemented the algorithm for Shannon base pairing entropy calculation into a program named TRIPLE. We tested it on ncRNA datasets and compared its performance on these ncRNAs with the performance achieved by the software NUPACK [23] and RNAfold [12,29] developed under the Boltzmann standard secondary structure ensemble [17,22].

### Data preparation

We downloaded the 13 ncRNA datasets previously investigated in Table 1 of [18]. They are of diverse functions, including pre-cursor microRNAs, group I and II introns, RNase P and MRP, bacterial and eukaryotic signal recognition particle (SRP), ribosomal RNAs, small nuclear spliceosomal RNAs, riboswitches, tmRNAs, regulatory RNAs, tRNAs, telomerase RNAs, small nucleolar RNAs, and Hammerhead ribozymes.

The results from using these datasets were analyzed with 6 different types of measures, including Z-score and *p*-value of minimal free energy (MFE), and Shannon base pairing entropy [18], in comparisons with random sequences. The six measures correlate to varying degrees, hence using MFE Z-score and Shannon base pairing entropy may be sufficient to cover the other measures. However, these two measures, as the respective indicators for the fold stability and fold certainty of ncRNA secondary structure, have varying performances on the 13 ncRNA datasets.

For our tests, we also generated random sequences as control data. For every ncRNA sequence, we randomly shuffled it to produce two sets of 100 random sequences each; one set was based upon single nucleotide shuffling, the other was based upon di-nucleotide shuffling. In addition, all ncRNA sequences containing nucleotides other than A, C, G, T, and U were removed for the reason that NUPACK [23] doesn't accept sequences containing wildcard symbols.

### Shannon entropy distribution of random sequences

Two energy model based softwares, NUPACK (with the pseudoknot function turned off) and RNAfold, and our program TRIPLE computed base pairing probabilities on ncRNA sequences and on random sequences. In particular, for every ncRNA sequence **x **and its associated randomly shuffled sequence set *S***_x_**, the Shannon entropies of these sequences were computed.

A Kolmogorov-Smirnov test (KS test) [24] was applied to verify the normality of the entropy distributions from all randomly shuffled sequence sets. The results show that for 99% of the sequence sets we fail to reject the hypothesis that entropies are normally distributed with 95% confidence level. This indicates that we may use a Z-score to measure performance.

### Z-score scores and comparisons

**Figure 1:**
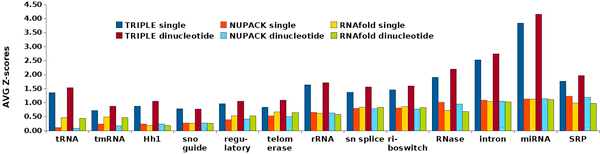
**Comparisons of averaged Z-score of Shannon base pairing entropies**. Comparisons of averaged Z-score of Shannon base pairing entropies computed by NUPACK, RNAfold, and TRIPLE for each of the 13 ncRNA datasets downloaded from [18].

For each ncRNA, the average and standard deviation of Shannon entropies of the randomly shuffled sequences were estimated. The Z-score of the Shannon entropy *Q*(**x**) of ncRNA sequence **x **is defined as follows:

(1) $$Z\text{(}x)=\frac{\mu (Q(S_{x}\text{))–}Q(x)}{\sigma (Q(S_{x}\text{))}}$$

where *μ*(*Q*(*S***_x_**)) and *σ*(*Q*(*S***_x_**)) respectively denote the average and standard deviation of the Shannon entropies of the random sequences in set *S***_x_**. The Z-Score measures how well entropies may distinguish the real ncRNA sequence **x **from their corresponding randomly shuffled sequences in *S***_x_**. Figure 1 compares the averages of the Z-scores of Shannon base pairing entropies computed by NUPACK, RNAfold, and TRIPLE on each of the 13 ncRNA datasets. It shows that TRIPLE significantly improved the Z-scores over NUPACK and RNAfold across all the 13 datasets.

To examine how the Z-scores might have been improved by TRIPLE, we designated four thresholds for Z-scores, which are 2, 1.5, 1, and 0.5. The percentages of sequences of each dataset with Z-score greater than or equal to the thresholds were computed.

Table 1 shows details of the Z-score improvements over NUPACK when di-nucleotide shuffling was used. With a threshold 2 or 1.5, our method performed better than NUPACK in all datasets. With the threshold 1 and 0.5, our method improved upon NUPACK in 12 and 10 datasets, respectively. The results of TRIPLE and NUPACK using a single nucleotide random shuffling are given in Table 2, which shows that our method also performs better than NUPACK in the majority of datasets. In particular, TRIPLE performed better than NUPACK in all datasets with threshold of 2; with threshold equal to 1.5 or 1, our method had better results than NUPACK in 12 datasets and in 9 datasets with threshold equal of 0.5.

**Table 1:** Comparisons of TRIPLE and NUPACK by the percentages of sequences falling in each category of a *Z*-score range.

ncRNA	Method	Z ≥ 2.0	Z ≥ 1.5	Z ≥ 1.0	Z ≥ 0.5
Hh1	TRIPLE	26.67	40.00	53.33	73.33
	NUPACK	0.00	0.00	20.00	53.33
sno_guide	TRIPLE	14.43	24.45	38.39	58.19
	NUPACK	0.73	8.80	27.63	45.23
sn_splice	TRIPLE	40.51	50.63	60.76	65.82
	NUPACK	3.80	18.99	48.10	70.89
SRP	TRIPLE	35.06	44.16	59.74	67.53
	NUPACK	3.90	36.36	72.73	85.71
tRNA	TRIPLE	29.56	51.33	70.97	86.02
	NUPACK	0.00	2.30	12.04	32.21
intron	TRIPLE	60.75	69.16	78.50	85.98
	NUPACK	1.87	19.63	61.68	85.05
riboswitch	TRIPLE	34.64	48.37	60.13	78.43
	NUPACK	1.96	18.95	45.75	69.28
miRNA	TRIPLE	81.48	88.89	94.07	97.04
	NUPACK	0.00	12.59	68.15	97.78
telomerase	TRIPLE	29.41	35.29	41.18	58.82
	NUPACK	11.76	17.65	35.29	47.06
RNase	TRIPLE	50.70	70.42	81.69	92.25
	NUPACK	5.63	23.94	48.59	72.54
regulatory	TRIPLE	22.41	24.14	32.76	56.90
	NUPACK	1.72	3.45	18.97	51.72
tmRNA	TRIPLE	18.64	32.20	45.76	55.93
	NUPACK	1.69	8.47	27.12	37.29
rRNA	TRIPLE	36.16	50.62	70.87	83.06
	NUPACK	4.75	21.07	42.56	61.16

Random sequences were obtained with di-nucleotide shuffling of the real ncRNA sequences.

**Table 2:** Comparisons of TRIPLE and NUPACK by the percentages of sequences falling in each category of a *Z*-score range.

ncRNA	Method	Z ≥ 2	Z ≥ 1.5	Z ≥1	Z ≥ 0.5
Hh1	TRIPLE	6.67	33.33	53.33	73.33
	NUPACK	0.00	0.00	20.00	60.00
sno_guide	TRIPLE	14.91	25.43	41.10	57.95
	NUPACK	0.98	9.05	28.85	45.72
sn_splice	TRIPLE	31.65	43.04	56.96	65.82
	NUPACK	5.06	26.58	51.90	69.62
SRP	TRIPLE	32.47	45.45	55.84	68.83
	NUPACK	3.90	37.66	72.73	87.01
tRNA	TRIPLE	24.07	45.31	64.25	79.47
	NUPACK	0.00	2.12	14.69	33.45
intron	TRIPLE	59.81	68.22	74.77	84.11
	NUPACK	1.87	22.43	66.36	85.98
riboswitch	TRIPLE	32.03	44.44	56.86	71.90
	NUPACK	1.96	21.57	46.41	69.28
miRNA	TRIPLE	75.56	81.48	90.37	93.33
	NUPACK	0.00	9.63	70.37	98.52
telomerase	TRIPLE	23.53	29.41	41.18	58.82
	NUPACK	5.88	29.41	29.41	52.94
RNase	TRIPLE	38.03	56.34	72.54	87.32
	NUPACK	10.56	26.06	52.11	76.06
regulatory	TRIPLE	18.97	25.86	31.03	51.72
	NUPACK	0.00	1.72	24.14	50.00
tmRNA	TRIPLE	15.25	27.12	38.98	57.63
	NUPACK	3.39	6.78	27.12	42.37
rRNA	TRIPLE	34.09	47.31	64.88	79.96
	NUPACK	6.40	21.69	43.19	60.74

Random sequences were obtained with single nucleotide shuffling of the real ncRNA sequences.

The results of RNAfold using the default setting are given in Table 3 and 4. Table 3 shows results on di-nucleotide shuffling datasets. TRIPLE works better in the majority of datasets. It outperforms RNAfold in all datasets with threshold equal to 2 and 1.5. With threshold of 1 and 0.5, TRIPLE wins 12 (tie 1) and 8 (tie 1) datasets, respectively. In Table 4, TRIPLE shows similar performance on single nucleotide shuffling datasets. It has better scores than RNAfold in 13, 13, 11, and 7 (tie 1) datasets with threshold of 2, 1.5, 1, and 0.5, respectively. In addition, RNAfold was tested with the available program options (tables not shown). With option "noLP" on RNAfold, TRIPLE performs better in 13, 13, 11 (tie 1), and 9 di-nucleotide shuffling datasets in terms of threshold of 2, 1.5, 1, and 0.5, respectively. In single nucleotide shuffling datasets, TRIPLE wins 13, 13, 12 and 8 datasets separately with threshold of 2, 1.5, 1, and 0.5.

**Table 3:** Comparisons of TRIPLE and RNAfold by the percentages of sequences falling in each category of a *Z*-score range.

Dataset	Method	≥2 (%)	≥1.5 (%)	≥1(%)	≥0.5 (%)
Hh1	TRIPLE	26.67	40.00	53.33	73.33
	RNAfold	0.00	0.00	20.00	53.33
sno_guide	TRIPLE	14.43	24.45	38.39	58.19
	RNAfold	1.71	7.82	23.96	43.03
sn_splice	TRIPLE	40.51	50.63	60.76	65.82
	RNAfold	6.33	21.52	54.43	69.62
SRP	TRIPLE	35.06	44.16	59.74	67.53
	RNAfold	5.19	24.68	58.44	71.43
tRNA	TRIPLE	29.56	51.33	70.97	86.02
	RNAfold	0.18	4.25	24.78	47.96
intron	TRIPLE	60.75	69.16	78.50	85.98
	RNAfold	2.80	17.76	60.75	84.11
riboswitch	TRIPLE	34.64	48.37	60.13	78.43
	RNAfold	0.65	17.65	47.06	70.59
miRNA	TRIPLE	81.48	88.89	94.07	97.04
	RNAfold	0.00	7.41	65.93	97.78
telomerase	TRIPLE	29.41	35.29	41.18	58.82
	RNAfold	0.00	23.53	41.18	58.82
RNase	TRIPLE	50.70	70.42	81.69	92.25
	RNAfold	1.41	12.68	34.51	59.15
regulatory	TRIPLE	22.41	24.14	32.76	56.90
	RNAfold	0.00	6.90	27.59	63.79
tmRNA	TRIPLE	18.64	32.20	45.76	55.93
	RNAfold	1.69	10.17	33.90	50.85
rRNA	TRIPLE	36.16	50.62	70.87	83.06
	RNAfold	1.45	15.70	35.33	56.82

Random sequences were obtained with di-nucleotide shuffling of the real ncRNA sequences.

**Table 4:** Comparisons of TRIPLE and RNAfold by the percentages of sequences falling in each category of a *Z*-score range.

Dataset	Method	≥2 (%)	≥1.5 (%)	≥1 (%)	≥0.5 (%)
Hh1	TRIPLE	6.67	33.33	53.33	73.33
	RNAfold	0.00	0.00	20.00	53.33
sno_guide	TRIPLE	14.91	25.43	41.10	57.95
	RNAfold	1.47	7.33	24.21	44.01
sn_splice	TRIPLE	31.65	43.04	56.96	65.82
	RNAfold	6.33	24.05	53.16	68.35
SRP	TRIPLE	32.47	45.45	55.84	68.83
	RNAfold	5.19	29.87	59.74	77.92
tRNA	TRIPLE	24.07	45.31	64.25	79.47
	RNAfold	0.00	6.19	26.19	48.85
intron	TRIPLE	59.81	68.22	74.77	84.11
	RNAfold	1.87	16.82	58.88	85.98
riboswitch	TRIPLE	32.03	44.44	56.86	71.90
	RNAfold	1.31	20.92	49.67	71.24
miRNA	TRIPLE	75.56	81.48	90.37	93.33
	RNAfold	0.74	10.37	69.63	97.78
telomerase	TRIPLE	23.53	29.41	41.18	58.82
	RNAfold	5.88	17.65	35.29	58.82
RNase	TRIPLE	38.03	56.34	72.54	87.32
	RNAfold	1.41	15.49	35.92	61.27
regulatory	TRIPLE	18.97	25.86	31.03	51.72
	RNAfold	0.00	5.17	32.76	67.24
tmRNA	TRIPLE	15.25	27.12	38.98	57.63
	RNAfold	0.00	11.86	35.59	45.76
rRNA	TRIPLE	34.09	47.31	64.88	79.96
	RNAfold	1.86	17.98	37.60	57.64

Random sequences were obtained with single nucleotide shuffling of the real ncRNA sequences.

When we specify "noLP" and "noCloseGU" on RNAfold, TRIPLE beats RNAfold in 13, 13, 12, and 11 di-nucleotide shuffling datasets, and 13, 13, 13, and 11 single nucleotide shuffling datasets with threshold 2, 1.5, 1, and 0.5, respectively. If we specify "noLP" and "noGU" on RNAfold, our method performs better on all di-nucleotide shuffling and single nucleotide shuffling datasets with all four thresholds.

We also compared TRIPLE, NUPACK, and RNAfold on some real genome background tests. Several genome sequences from bacteria, archaea, and eukaryotes were retrieved from the NCBI database. Using these genome sequences, we created genome backgrounds for the 13 ncRNA data sets. In particular, for each RNA sequence from 13 ncRNA data sets, 100 sequence segments of the same length were sampled from each genome sequence and used to test against the RNA sequence to calculate base pairing entropies and Z-score. With such genome backgrounds, the overall performance of TRIPLE on the 13 ncRNA data sets is mixed and is close to that of NUPACK and RNAfold (data not shown). This performance of TRIPLE on real genomes indicates that there is still a gap between the ability of our method and successful ncRNA gene finding. Nevertheless, the test results reveal that the constrained "triple base pairs" model is necessary but still not sufficient enough. This suggests incorporating further structural constraints will improve the effectiveness for ncRNA search on real genomes.

To roughly evaluate the speed of the three tools, the running time for 101 sequences, including 1 real miRNA sequence and its 100 single nucleotide shuffled sequences, was measured on a Linux machine with an Intel dual-core CPU (E7500 2.93 GHz). Each sequence has 100 nucleotides. TRIPLE, NUPACK, and RNAfold spent 20.7 seconds, 36.2 seconds and 3.4 seconds, respectively. We point out that TRIPLE has the potential to be optimized for each specific grammar to improves its efficiency.

## Discussion

This work introduced a modified ensemble of ncRNA secondary structures with the constraint of requiring only canonical base pairs to only occur and that stems must be energetically stable in all the alternative structures. The comparisons of performances between our program TRIPLE and energy model based software (NUPACK and RNAfold) implemented based on the canonical structure ensemble have demonstrated a significant improvement in the entropy measure for ncRNA fold certainty by our model. In particular, an improvement of the entropy Z-scores was shown across almost all 13 tested ncRNAs datasets previously used to test various ncRNA measures [18].

We note that there is only one exceptional case observed from Table 1, 2, 3, 4: SRP whose entropy Z-score performance was not improved (as much as other ncRNAs) when *Z <*1.5. The problem might have been caused by the implementation technique rather than the methodology. Most of the tested SRP RNA sequences (Eukaryotic and archaeal 7S RNAs) are of length around 300 and contain about a dozen stems. In many of them, consecutive base pairs are broken by internal loops into small stem pieces, some having only two consecutive canonical pairs; whereas, in our SCFG implementation we simply required three consecutive base pairs as a must in a stem, possibly missing the secondary structure of many of these sequences. This issue with the SCFG can be easily fixed, e.g., by replacing the SCFG with one that better represents the constrained Boltzmann ensemble in which stems are all energetically stable.

To ensure that the performance difference between TRIPLE and energy model based software (NUPACK and RNAfold) was *not *due to the difference in the thermodynamic energy model (Boltzmann ensemble) and the simple statistical model (SCFG) with stacking rules, we also constructed two additional SCFG models, one for unconstrained base pairs and another requiring at least two consecutive canonical base pairs in stems. Tests on these two models over the 13 ncRNA data set resulted in entropy Z-scores (data not shown) comparable to those obtained by NUPACK and RNAfold but inferior to the performance of TRIPLE. We attribute the impressive performance by TRIPLE to the constraint of "triple base pairs" satisfied by real ncRNA sequences but which is hard to achieve for random sequences.

Since the entropy Z-score improvement by our method was not uniform across the 13 ncRNAs, one may want to look into additional other factors that might have contributed to the under-performance of certain ncRNAs. For example, the averaged GC contents are different in these 13 datasets, with SRP RNAs having 58% GC and standard deviation of 10.4%. A sequence with a high GC content is more likely to produce more spurious, alternative structures, possibly resulting in a higher base pairing entropy. However, since randomly shuffled sequences would also have the same GC content, it becomes very difficult to determine if the entropies of these sequences have been considerably affected by the GC bias. Indeed, previous investigations [25] have revealed that, while the base composition of a ncRNA is related to the phylogenetic branches on which the specific ncRNA may be placed, it may not fully explain the diverse performances of structure measures on various ncRNAs. Notably it has been discovered that base compositions are distinct in different parts of rRNA secondary structure (stems, loops, bulges, and junctions) [26], suggesting that an averaged base composition may not suitably represent the global structural behavior of an ncRNA sequence.

Technically the TRIPLE program was implemented with an SCFG that assumes stems to have at least three consecutive canonical base pairs. Yet, as we pointed out earlier, the performance results should hold for a constrained Boltzmann ensemble in which stems are required to be energetically stable. This constraint of stable stems was intended to capture the energetic stability of helical structures in the native tertiary fold [27,28]. Since the ultimate distinction between a ncRNA and a random sequence lies in its function (thus tertiary structure); additional, critical tertiary characteristics may be incorporated into the structure ensemble to further improve the fold certainty measure. In our testing of stem stability (see section "Energetically stable stems"), ncRNA sequences from the 51 datasets demonstrated certain sequential properties that may characterize tertiary interactions, e.g., coaxial stacking of helices. However, to computationally model tertiary interactions, a model beyond a context-free system would be necessary; thus it would be difficult to use an SCFG or a Boltzmann ensemble for this purpose. We need to develop methods to identify tertiary contributions critical to the Shannon base pairing entropy measure and to model such contributions. Although this method and technique have been developed with reference to non-coding RNAs, it is possible that protein-coding mRNAs would display similar properties, when sufficient structural information about them has been gathered.

## Conclusions

We present work developing structure measures that can effectively distinguish ncRNAs from random sequences. We compute Shannon base pairing entropies based on a constrained secondary structure model that favors tertiary folding. Experimental results indicate that our approach significantly improves the Z-score of base pairing Shannon entropies on 13 ncRNA datasets [18] in comparison to that computed by NUPACK [23] and RNAfold [12,29]. These results shows that investigating secondary structure ensembles of ncRNAs is helpful for developing effective structure-based ncRNA gene finding methods.

## Method and model

Our method to distinguish ncRNAs from random sequences is based on measuring of the base pairing Shannon entropy [15,16] under a new RNA secondary structure model. The building blocks of this model are stems arranged in parallel and nested patterns connected by unpaired strand segments, similar to those permitted by a standard ensemble [11,17,29]. The new model is constrained, however, to contain a smaller space of equilibrium alternative structures, requiring there are only energetically stable stems (e.g., of free energy levels under a threshold) to occur in the structures. The constraint is basically to consider the effect of energetically stable stems on tertiary folding and to remove spurious structures that may not correspond to a tertiary fold. According to the RNA folding pathway theory and the hierarchical folding model [27,28,30], building block helices are first stabilized by canonical base pairings before being arranged to interact with each other or with unpaired strands through tertiary motifs (non-canonical nucleotide interactions). A typical example is the multi-loop junctions in which one or more pairs of coaxially stacked helices bring three or more regions together, further stabilized by the tertiary motifs at the junctions [31,32]. The helices involved are stable before the junction is formed or any possible nucleotide interaction modifications are made to the helical base pairs at the junction [33].

### Energetically stable stems

**Figure 2:**
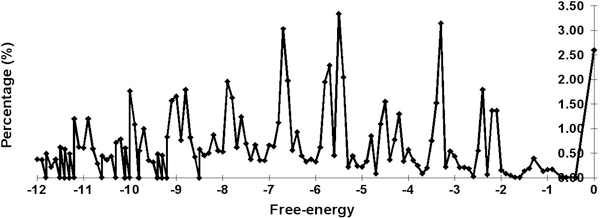
**Percentages of free-energy of stems**. Percentages of free-energy of stems from 51 Rfam datasets (percentages of stems with free-energy less than -12 are not given in this figure).

**Figure 3:**
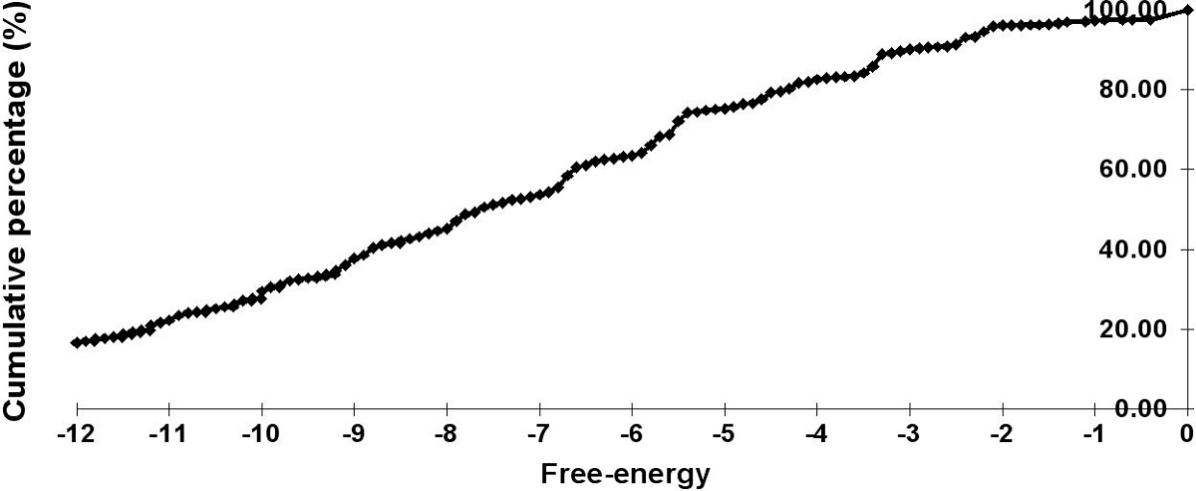
**Cumulative percentages of free-energy of stems**. Cumulative percentages of free-energy of stems from 51 Rfam datasets (cumulative percentages of stems with free-energy less than -12 are not given in this figure). Note the step at -3.4.

A stem is the atomic, structural unit of the new secondary structure space. To identify the energy levels of stems suitable to be included in this model, we conducted a survey on the 51 sets of ncRNA seed alignments, representatives of the ncRNAs in Rfam [34], which had been used with the software Infernal [35] as benchmarks. From each ncRNA seed structural alignment, we computed the thermodynamic free energy of every instance of a stem in the alignment data using various functions of the Vienna Package [12,29] as follows. RNAduplex was first applied to the two strands of the stem marked by the annotation to predict the optimal base pairings within the stem, then, the minimum free energy of the predicted stem structure, with overhangs removed, was computed with RNAeval. Figures 2 and 3 respectively show plots of the percentages and cumulative percentages of free energy levels of stems in these 51 ncRNA seed alignments.

The peaks (with relatively high percentages) on the percentage curve of Figure 2 indicate concentrations of certain types of stems at energies levels around -4.5, -3.3, and -2.4 kcal/mol. Since a G-U pair is counted weakly towards the free energy contribution (by the Vienna package), we identified the peak value -4.5 kcal/mol to be the free energy of stems of three base pairs, with two G-C pairs and one A-U in the middle or two A-U pairs and one G-C in the middle. The value -3.3 kcal/mol is the free energy of stems containing exactly two G-C pairs or stems with one G-C pair followed by two A-U pairs. Values around -2.4 kcal/mol are stems containing one G-C and an A-U pair or simply four A-U pairs.

Based on this survey, we were able to identify two energy thresholds: -3.4 and -4.6 kcal/mol for *semi-stable stems *and *stable stems *respectively. Both require at least three base pairs of which at least one is G-C pair. We further observed the difference between these two categories of stems on the 51 ncRNA datasets. In general, although levels of energy appear to be somewhat uniformly distributed (see Figure 3), an overwhelmingly large percentage of stems in both categories are located in the vicinity of other stems. In particular, 79.6% of stable stems (with a free energy -4.6 kcal/mol or lower) have 0 (number of nucleotides) distance from their closest neighbor stem and 16.5% of stable stems have distance 1 from their closest neighbors. For semi-stable stems, the group having zero distance to other stems is 85.6% of the total while the group having distance 1 is 10.6%. Since zero distance between two stems may reflect a contiguous strand connecting two coaxially stacked helices in tertiary structure, our survey suggests a semi-stable stem interacts with another stem to maintain even its own local stability. In the rest of this work, we do not distinguish between stable and semi-stable stems. In conducting this survey, we did not directly use the stem structures annotated in the seed alignments to compute their energies. Due to evolution, substantial structural variation may occur across species; one stem may be present in one sequence and absent in another but a structural alignment algorithm may try to align all sequences to the consensus stem, giving rise to "misalignments" which we have observed [36]. Most of such "malformed stems" mistakenly aligned to the consensus often contain bulges or internal loops and have higher free energies greater than the threshold -3.4 kcal/mol.

### The RNA secondary structure model

In the present study, a secondary structure model is defined with a Stochastic Context Free Grammar (SCFG) [37]. Our model requires there are at least three consecutive base pairs in every stem; the constraint is described with the following seven generic production rules:

(1) *X *→ *a *(2) *X *→ *aX *(3) *X *→ *aHb*

(4) *X *→ *aHbX *(5) *H *→ *aHb *(6) *H *→ *aYb *

(7) *Y *→ *aXb*

where capital letters are non-terminal symbols that define substructures and low case letters are terminals, each being one of the four nucleotides A, C, G, and U.

The starting non-terminal, *X*, can generate an unpaired nucleotide or a base pair with the first three rules. The fourth rule generates two parallel substructures. Non-terminal *H *is used to generate consecutive base pairs with non-terminal *Y *to generate the closing base pair. Essentially, the process of generating a stem needs to recursively call production rules with the left-hand-side non-terminals *X*, *H *and *Y *each at least once. This constraint guarantees that every stem has at least three consecutive base pairs, as required by our secondary structure model.

### Probability parameter calculation

There are two sets of probability parameters associated with the induced SCFG. First, we used a simple scheme of probability settings for the unpaired bases and base pairs, with a uniform 0.25 probability for every base. The probability distribution of {0.25, 0.25, 0.17, 0.17, 0.08, 0.08} is given to the six canonical base pairs G-C, C-G, A-U, U-A, G-U, and U-G; a probability of zero is given to all non-canonical base pairs. Alternatively, probabilities for unpaired bases and base pairs may be estimated from available RNA datasets with known secondary structures [34], as has been done in some of the previously work with SCFGs [38,39].

Second, we computed the probabilities for the production rules of the model as follows. To allow our method to be applicable to all structural ncRNAs, we did not estimate the probabilities based on a training data set. In fact, we believe that the probability parameter setting of an SCFG for the fold certainty measure should be different from that for fold stability measure (i.e., folding). Based on the principle of maximum entropy, we developed the following approach to calculate the probabilities for the rules in our SCFG model.

Let *p_i _*be the probability associated with the production rule *i*, for *i *= 1, 2,...,7, respectively. Since the summation of probabilities of rules with the same non-terminal on the left-hand-side is required to be 1, we can establish the following equations:

$$\left\{\begin{array}{cc} p_{1}+p_{2}+p_{3}+p_{4} & =1\\ p_{5}+p_{6} & =1\\ p_{7} & =1 \end{array}\right)$$

Let

$$q_{bp}=\sqrt[{6}]{0.25\times 0.25\times 0.17\times 0.17\times 0.08\times 0.08}$$

be the geometric average of the six base pair probabilities. According to the principle of maximum entropy, given we have no prior knowledge of a probability distribution, the assumption of a distribution with the maximum entropy is the best choice, since it will take the smallest risk [40]. If we apply this principle to our problem, the probability contribution from a base pair should be close to the contribution from unpaired bases. Rule probabilities can be estimated to satisfy following equations:

$$\left\{\begin{array}{cc} p_{1} & =p_{2}\\ p_{3} & =p_{4}\\ {\left(q_{bp}\right)}^{3}\times p_{3}\times p_{6}\times p_{7} & ={\left(0.25\times p_{1}\right)}^{6}\\ {\left(q_{bp}\right)}^{4}\times p_{3}\times p_{5}\times p_{6}\times p_{7} & ={\left(0.25\times p_{1}\right)}^{8} \end{array}\right)$$

From above equations, it follows that

$$\begin{array}{ccc} p_{1}=0.499 & p_{2}=0.499 & p_{3}=0.001\\ p_{4}=0.001 & p_{5}=0.103 & p_{6}=0.897\\ p_{7}=1 & & \end{array}$$

### Computing base pairing Shannon entropy

Based on the new RNA secondary structure model, we can compute the fold certainty of any given RNA sequence, which is defined as the Shannon entropy measured on base pairings formed by the sequence over the specified secondary structure space Ω. Specifically, let the sequence be *x *= *x*_1_*x*_2 _... *x_n _*of *n *nucleotides. For indexes *i < j*, the probability *P_i,j _*of base pairing between bases *x_i _*and *x_j _*is computed with

(2) $$P_{i,j}\left(x\right)=\underset{s\in \Omega }{\sum }p\left(s,x\right)\delta {\left(x\right)}_{i,j}^{s}$$

where *p*(*s*, *x*) is the probability of *x *being folded into to the structure *s *in the space Ω and $\delta {\left(x\right)}_{i,j}^{s}$ is a binary value indicator for the occurrence of base pair (*x_i_*, *x_j_*) in structure *s*. The Shannon entropy of *P_i,j_*(*x*) is computed as [15,16]

(3) $$Q\left(x\right)=-\frac{1}{n}\underset{i<j}{\sum }P_{i,j}\left(x\right)\text{log}P_{i,j}\left(x\right)$$

To compute the expected frequency of the base pairing, *P_i,j_*(*x*), with formula (2), we take advantage of the Inside and Outside algorithms developed for SCFG [37]. Given any nonterminal symbol *S *in the grammar, the *inside probability *is defined as

$$\;\alpha \left(S,i,j,x\right)=Prob\left(S\Rightarrow^{*}x_{i}x_{i+1}\cdots x_{j}\right)$$

i.e., the total probability for the sequence segment *x_i_x_i_*_+1 _... *x_j _*to adopt alternative substructures specified by *S*. Assume *S*_0 _to be the initial nonterminal symbol for the SCFG model. Then *α*(*S*_0_, 1, *n*, *x*) is the total probability of the sequence *x*'s folding under the model.

**Figure 4:**
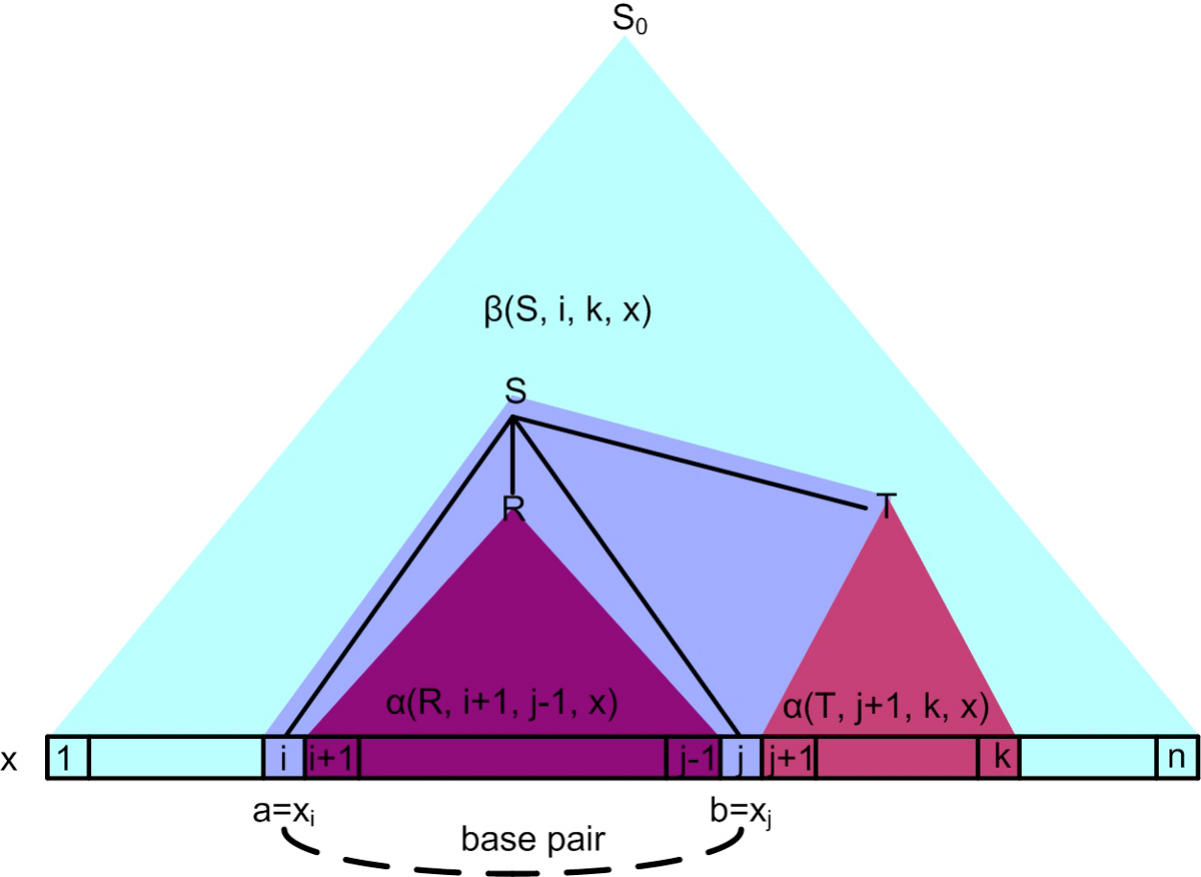
**Illustration of the application of the generic production rule**. Illustration of the application of the generic production rule *S *→ *aRbT *that produces a base pair between positions *i *and *j *for the query sequence *x*, provided that the start non-terminal *S*_0 _derives *x*_1_*x*_2 _... *x_i_*_-1_*Sx_k_*_+1 _... *x_n_*. Note that given *i *and *j*, the position of *k *can vary.

The *outside probability *is defined as

$$\beta \left(S,i,j,x\right)=Prob\left(S_{0}\Rightarrow^{*}x_{1}\cdots x_{i-1}Sx_{j+1}\cdots x_{n}\right)$$

i.e., the total probability for the whole sequence *x*_1 _... *x_n _*to adopt all alternative substructures that allow the sequence segment from position *i *to position *j *to adopt any substructure specified by *S *(see Figure 4 for illustration).

*P_i,j_*(*x*) then can be computed as the normalized probability of the base pair (*x_i_*, *x_j_*) occurring in all valid alternative secondary structures of *x*:

(4) $$\frac{\underset{S\to aRbT}{\sum }Prob\left(S\to aRbT,a=x_{i},b=x_{j}\right)\gamma \left(R,S,T,i,j,x\right)}{\alpha \left(S_{0},1,n,x\right)}$$

where

$$\begin{array}{llll} \gamma \left(R,S,T,i,j,x\right) & =\underset{j<k\leq n}{\sum }\alpha \left(R,i+1,j-1,x\right)\; & & \;\\ & \times \beta \left(S,i,k,x\right)\times \alpha \left(T,j+1,k,x\right)\; & & \;\\ & \; & \end{array}$$

in which variables *S*, *R*, *T *are for non-terminals and variable production *S *→ *aRbT *represents rules (3)~(7) which involve base pair generations. For rules where *T *is empty, the summation and term α(*T*, *j *+ 1, *k*, *x*) do not exist and *k *is fixed as *j*.

The efficiency to compute *P_i,j_*(*x*) mostly depends on computing the *Inside *and *Outside *probabilities, which can be accomplished with dynamic programming and has the time complexity *O*(*mn*^3^) for a model of *m *nonterminals and rules and sequence length *n*.

## Competing interests

The authors declare that they have no competing interests.

## Authors' contributions

YW contributed to grammar design, algorithm development, program implementation, data acquisition, tests, result analysis, and manuscript drafting. AM contributed to algorithm design and program implementation. PS and TIS contributed to data acquisition and tests. YWL participated in model discussion. RLM contributed to the supervision, data acquisition, results analyses, biological insights, and manuscript drafting. LC conceived the overall model and algorithm and drafted the manuscript. All authors read and approved the manuscript.
